# Anxiety and Depression in Chronic Obstructive Pulmonary Disease: Perspectives on the Use of Hypnosis

**DOI:** 10.3389/fpsyg.2022.913406

**Published:** 2022-05-19

**Authors:** Hernán Anlló, François Larue, Bertrand Herer

**Affiliations:** ^1^Laboratory of Cognitive and Computational Neuroscience, Department of Cognitive Studies, École Normale Supérieure de Paris, PSL University, Paris, France; ^2^Complementary Care and Behavior Research Team, Bligny Hospital Center, Briis-sous-Forges, France; ^3^Palliative Care Unit, Bligny Hospital Center, Briis-sous-Forges, France; ^4^Pneumology Unit, Bligny Hospital Center, Briis-sous-Forges, France

**Keywords:** COPD, anxiety, depression, hypnosis, breathlessness, comorbidity, self-management, complementary care

## Abstract

Chronic Obstructive Pulmonary Disease (COPD) is a highly prevalent and debilitating respiratory condition, characterized by chronic airflow limitation, breathlessness, and other persistent respiratory symptoms. Critically, patients suffering from COPD often find themselves trapped in a vicious comorbidity cycle: while breathlessness and increased respiratory rate are known inducers of anxiety, the latter have been shown in turn to exacerbate breathlessness and chest discomfort. Hypnosis holds great potential for the simultaneous complementary management of anxiety and breathlessness in COPD. It is an inexpensive psychological intervention tailored to the patient’s own experience, convenient in terms of logistics and implementation. In this short qualitative review, we present hypnosis’ structural, cognitive, and neural fundamentals, and assess existing instances of hypnosis use in the treatment of anxiety, depression, and respiratory disease. We then discuss its potential as a tool for improving health-related quality of life and the self-management of COPD within (and beyond) pulmonary rehabilitation.

## Introduction

Chronic Obstructive Pulmonary Disease (COPD) causes persistent and progressive respiratory symptoms, including breathlessness, sputum, and suboptimal oxygenation (GOLD Report, 2022). Aside from a daunting mortality rate [3.23 million deaths in 2019 according to the WHO (2020) report on noncommunicable diseases], COPD hinders patients’ health-related quality of life (HRQoL) by reducing mobility, increasing fatigue levels, and propitiating psychological comorbidities such as anxiety, depression, and suicidality (Kellner et al., 1992; Hegerl and Mergl, 2014; Pumar et al., 2014). The 2022 edition of the Global Initiative for Obstructive Lung Disease report (GOLD) observes that treating psychological comorbidities is critical in COPD, as evidence shows that the alleviation of anxiety and depression symptoms also improves respiratory disease prognosis. In particular, the complementary use of cognitive behavioral therapy and mind–body interventions such as mindfulness-based therapy have been found to reliably reduce anxiety and depression in COPD, diminish fatigue, and improve lung function and exercise capacity (Farver-Vestergaard et al., 2015).

Evidence shows that hypnosis is a fast, cost-effective intervention for the treatment of anxiety and depression, both as stand-alone therapy and as a part of larger therapeutic strategies (Hammond, 2010; Cafarella et al., 2012; Milling et al., 2019; Valentine et al., 2019). On the grounds of its implementational and therapeutic advantages, it is worth discussing its incorporation to the treatment of breathlessness-related anxiety and depression in COPD. In the present work, we succinctly introduce hypnosis’ structure, its cognitive building blocks, and its basic neural correlates. We then reflect upon how hypnosis could contribute to the treatment of transient and chronic anxiety and depression in COPD, and its compatibility with pulmonary rehabilitation and self-management strategies.

## COPD, Psychological Comorbidities, and Quality of Life

At the current juncture, COPD is a chronic, incurable condition. This makes improving patients’ symptoms and HRQoL a chief priority in the management of the disease (Engström et al., 2001). Because of its handicapping nature, COPD progression affects all subjective and objective dimensions of HRQoL in an incremental fashion (Afroz et al., 2020). On the physical level, it restricts general physical function and breathing mechanics, leading to increased levels of fatigue and reduced autonomy. On the psychological level, it fosters negative affects, increases emotional burden and negative coping. On the social level, it restricts the patient’s capability to work and generally impacts interpersonal relations and autonomy.

While multiple comorbidities are associated with COPD, anxiety and depression have been reliably identified as some of the most important predictors of poor HRQoL and treatment adherence (Dalal et al., 2011; Willgoss and Yohannes, 2013; Yohannes and Alexopoulos, 2014). Timely diagnosing anxiety and depression in COPD have proven particularly challenging due to symptom overlapping and an unclear etiological association between conditions (Pumar et al., 2014). However, identification and treatment of these psychological comorbidities are paramount: evidence shows that anxiety, depression, and suicidality are not only highly prevalent among the COPD population (Kunik et al., 2005; Stage et al., 2006; Sampaio et al., 2019), but also are accurate predictors of poorer health status, of increased risk of exacerbation, and of higher emergency admissions (Blakemore et al., 2019). Studies exploring HRQoL in stable and severe COPD cohorts have found clear associations between anxiety and depression levels, and poorer quality of life (Cully et al., 2006; Eisner et al., 2010). This is unsurprising, given the vicious bidirectional nature of the relationship between COPD and these psychological comorbidities (Atlantis et al., 2013). On the one hand, breathlessness, chest tightness, and increased respiratory rate are known inducers of anxiety (Kellner et al., 1992; Tselebis et al., 2016), and the psychosocial adversity caused by COPD can easily lead to depression (Alexopoulos, 2005). On the other hand, anxiety and depression are common culprits for the acute worsening of chronic breathlessness, chest pain, fatigue, and other prominent COPD symptoms (Atlantis et al., 2013; Simon et al., 2013).

Interestingly, clinical and biological markers of COPD appear to be less important determinants of depression than actual feelings of breathlessness and subjective appreciation of HRQoL (Hanania et al., 2011). At the same time, the impact of anxiety and depression on HRQoL in COPD appears to be decorrelated from bronchiectasis and objective lung function (Engström et al., 2001; Ekici et al., 2015). Overall, these findings suggest that improvements of HRQoL in COPD may depend on therapeutic strategies that concentrate on patients’ subjective and experiential correlates of the disease, or at the very least takes them seriously into account.

## Hypnosis Fundamentals

To the effects of the present review, it is more convenient to privilege a procedural definition of hypnosis rather than to navigate the long-standing theoretical debates on the nature of the phenomenon (Terhune, 2014; Terhune et al., 2017). During a standard hypnotic intervention, customarily a trained professional (e.g., a researcher, a medical doctor, and a therapist) delivers a suggestion (e.g., motor, cognitive, and affective) to a receptor (e.g., the participant of a research protocol and a patient). Usually, this suggestion is preceded by an induction phase composed of relaxation and attention exercises, aimed at producing experiential and motivational changes that serve the purpose of enhancing the receptor’s permeability to suggestion (Woody and Sadler, 2016). Much like placebo interventions, hypnotic interventions work best when practiced within a socio-cultural context that increases the receptor’s motivation and compliance (e.g., a lab and a hospital; Lynn and Sherman, 2000).

**Figure 1:**
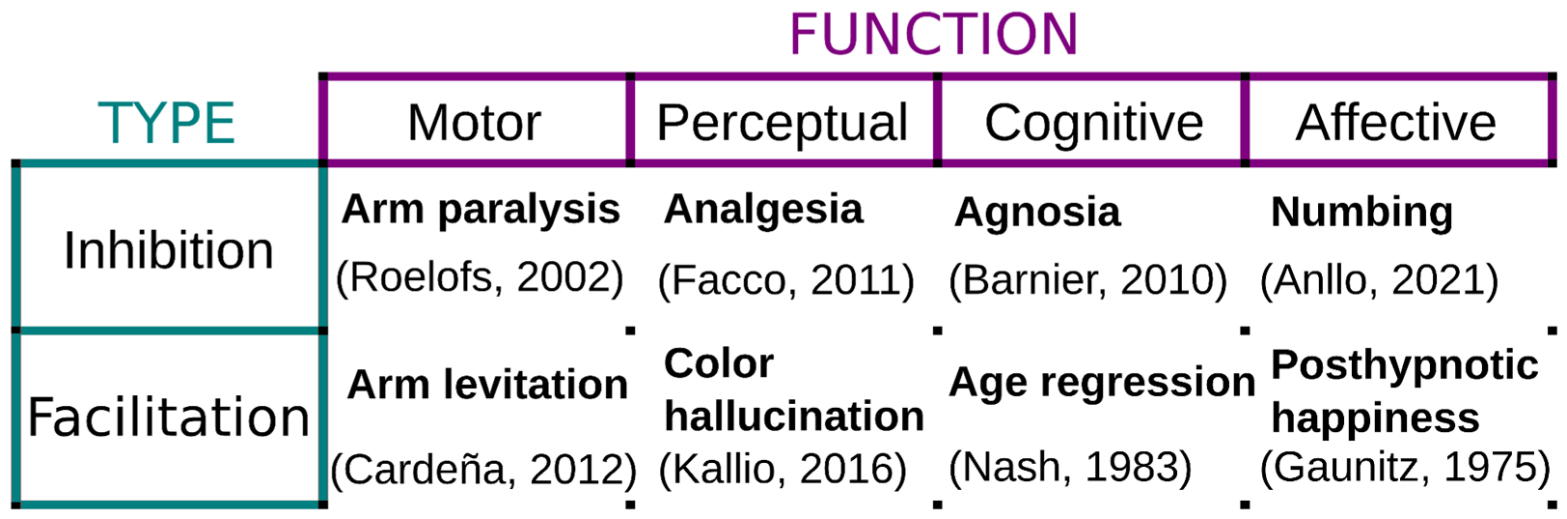
Examples of hypnotic suggestions. Hypnotic and posthypnotic suggestions can either inhibit or facilitate a vast array of motor, perceptual, cognitive, and affective responses. Examples are provided together with studies evaluating their implementation and phenomenology.

When performed under these conditions, hypnosis elicits a hypnotic response, which consists of the inhibition/facilitation of all sorts of motor, sensory, cognitive, or affective responses. For example, an inhibitory motor suggestion can successfully induce paralysis and set off neurophysiological patterns different from simulated paralysis (Cojan et al., 2009). On the other hand, a facilitatory perceptual suggestion can effectively trigger hallucinatory content for susceptible individuals (Woody and Szechtman, 2011) or the onset of positive feelings (Gaunitz et al., 1975). Figure 1 below presents a short summary with examples of tested hypnotic suggestions, sorted by type, the function they target, and the hypnotic response they are known to produce.

While there is no definitive answer on what are the cognitive mechanisms behind hypnotic responses, reasonable consensus has been reached that they rely primarily on cognitive control and the top-down modulation of perception (Terhune et al., 2017). Perception is built simultaneously by bottom-up sensory information, and top-down conceptual information stemming from prior world-knowledge and expectations (De Lange et al., 2018). Crucially, it has been shown that, when purposefully managed, top-down influences can drastically shape perception (Carrasco et al., 2004; Balcetis and Dunning, 2006, 2010). Hypnosis would thus be a particularly powerful technique for the maximization of the top-down influence in the building of perceptual experience: instead of accessing the usual perceptual priors triggered by standard contextual information, hypnotized individuals rely on the hypnotic mental representations and expectations conveyed to them through hypnotic suggestion and use them to consolidate an alternate perceptual experience instead (Brown and Oakley, 2004).

Concerning hypnosis’ neural substrates, much remains to be uncovered. Yet, several studies coincide in pointing out common brain correlates to hypnotic responding, such as (1) reduced activity in the dorsal Anterior Cingulate Cortex, (2) increased functional connectivity between the dorsolateral prefrontal cortex and the insula in the Salience Network (Jiang et al., 2017), and (3) reduced connectivity between the Executive Control Network and the Default-Mode Network (Jiang et al., 2017; Landry et al., 2017). At the neurophysiological level, Jensen et al. (2015) have proposed that the changes detected in theta oscillations during hypnosis may act as facilitators of hypnotic responding.

Hypnosis has long been used with diverse therapeutic purposes, such as introducing and reinforcing better adaptive behavioral patterns (e.g., to diminish compulsory acting), thinking patterns (e.g., to counteract depressive ruminations), and emotional response (e.g., to induce calmness in the aftermath of trauma; Barabasz et al., 2010). Furthermore, hypnosis is of great use for re-orienting attention away from aversive stimuli, which has warranted it a particularly popular place in acute, chronic, and perioperative pain management therapies (Patterson and Jensen, 2003; Patterson et al., 2006; Patterson, 2010). It can yield positive results when utilized as stand-alone therapy but is most effective when implemented as a therapeutic complement to an already established psychological treatment or medical procedure (Ramondo et al., 2021). Oftentimes, practitioners wonder whether a hypnotic intervention’s efficacy will be conditioned to the patients’ hypnotic suggestibility. While, indeed, hypnotizability is the main predictor of successful hypnotic responding in experimental hypnosis (Barnier et al., 2021), suggestions posed in the context of medical treatments are generally easy to follow and do not demand a particularly high susceptibility (e.g., relaxation, searching for positive memories, and evoking mental imagery). Further, existing evidence has indicated that the success of hypnosis in the clinical milieu depends primarily on patient motivation and expectation (Barber, 1980), even when treating psychological conditions as complex as anxiety and depression (Yapko, 2001).

Finally, evidence shows that the technique is safe, and the risks associated with it (e.g., evoking bad memories and emotional abreactions) are negligible (Lynn et al., 1996). Peer-reviewed research on hypnosis safety suggests that the occurrence of “negative” sensations following hypnosis is rare, and decorrelated from suggestibility, which indicates hypnosis may not be at the source of these feelings to begin with (Brentar et al., 1992; Lynn et al., 1996).

## Hypnosis and the Complementary Management of Mood and Respiratory Disorders

Over the past 30 years, efforts to assess the efficacy of hypnosis as a therapeutic tool for the treatment of anxiety and depression in a controlled manner have progressively mounted, with favorable results. While less numerous, promising studies on the use of hypnosis in respiratory medicine have also shown that the technique can flexibly target key respiratory symptoms present in COPD.

A recent comprehensive meta-analysis including 13 randomly controlled trials (RCT) has assessed the efficacy of hypnosis for treating depression symptoms (Milling et al., 2019). Results show that hypnosis samples presented a mean significant improvement superior to controls, both at treatment end (effect size of improvement *d* = 0.71) and follow-up (*d* = 0.52). Such an impact places hypnosis within the same range of efficacy of other forms of treatment such as cognitive behavioral therapy (*d* = 0.67) and short-term psychodynamic therapy (*d* = 0.69; Cuijpers et al., 2011). Studies posterior to this meta-analysis continued to confirm this trend. For example, Fuhr et al. (2021) have shown no difference in mean reduction of depressive symptoms between hypnosis and cognitive behavioral therapy after 16–20 sessions, nor at the 6-month and 12-month follow-ups. In a study comparing hypnosis to meditation and progressive muscle relaxation in children with primary headaches, all three methods were shown to reliably reduce depression symptoms after 9 months of treatment (Jong et al., 2019). Aravena et al. (2020) also showed a significant decrease of depressive symptomatology after audio-recorded hypnosis sessions in patients with fibromyalgia.

The evaluation of evidence concerning the use of hypnosis for the management of anxiety is also positive overall. In a novel meta-analysis, Valentine et al. (2019) analyzed 17 RCT and found a mean significant improvement of anxiety against controls at treatment end (*d* = 0.79; *d* = 1.12 when contrasted against no-contact controls) and during follow-up (*d* = 0.99). Of note, hypnosis was at least as effective as cognitive behavioral therapy (*d* = 0.82) and better than mindfulness meditation (*d* = 0.39; Mitte, 2005; Blanck et al., 2018). Here as well, newer studies on the efficacy of hypnosis for treating anxiety symptoms as either stand-alone or complementary therapy indicate intervention effectiveness. To name a few, Roberts et al. (2021) have shown support for the use of hypnosis to reduce symptoms of anxiety among postmenopausal women. Roberts et al. (2021) indicate that hypnosis is a suitable adjunct in Crohn’s disease and may improve general psychosocial QoL, including anxiety.

Concerning the use of hypnosis specifically targeted at respiratory diseases, evidence has been somewhat scarce, but equally promising. Hypnosis-based psychodynamic treatments were proven effective for reducing anxiety and depression in amyotrophic lateral sclerosis patients with impaired respiratory function (Kleinbub et al., 2015). Further examples include hypnosis for improving breathlessness in pediatric medicine (McBride et al., 2014), asthma (Brown, 2007), and palliative care (Brugnoli, 2016; Montgomery et al., 2017). While no meta-analysis has been conducted to date, the observed main benefits of incorporating hypnosis to the management of respiratory conditions include relief of anxiety related to ventilation problems, alleviation of discomfort, and improvements in breathing regulation (Anbar, 2012).

In the specific case of COPD, hypnosis has been used almost exclusively as a relaxation technique (Cafarella et al., 2012). To our knowledge, there is only one RCT evaluating the use of hypnosis to manage anxiety and breathlessness in COPD that implements a relaxation control (Anlló et al., 2020). This crossover study has shown that a 15-min scripted hypnotic intervention positively impacted respiratory rate, pulsated oxygen saturation, Borg scores, and anxiety (as assessed by State–Trait Anxiety Inventory – 6 items version).

Interestingly, as of 2022, many new trials assessing the impact of hypnotic interventions on depression and anxiety symptoms are currently in progress, which shows that the technique continues to accrue interest in the medical community (e.g., Anlló et al., 2021a; Grégoire et al., 2022; Fernandes et al., *ongoing* NCT04010825). In particular, at least two of these ongoing trials are targeting the use of hypnosis specifically for the psychological and emotional correlates of COPD (Anlló et al., 2021b; Fernandes et al., *ongoing* NCT04010825).

## Discussion

While hard to disentangle, it is important to understand the separate roles of each of the building blocks of hypnosis as an intervention. Treating the technique as a monolithic interventional battery hinders our understanding of its real potential in respiratory medicine. For example, when implemented in respiratory medicine, and particularly in COPD, hypnosis has been mostly used as a form of “relaxation therapy” (Cafarella et al., 2012; Tselebis et al., 2016). Certainly, hypnotic inductions often include relaxation exercises (Batty et al., 2006), and of course, the implementation of hypnosis and other forms of guided mental imagery as a form of relaxation is beneficial in and of itself (Hammond, 2010; Volpato et al., 2015, 2022). However, as explained above, hypnotic effects depend primarily on the contents of suggestions (Figure 1), which are fundamentally independent from hypnosis’ relaxation component (Cardeña et al., 2012). Thus, a different use of hypnosis, where the emphasis is shifted toward tailoring suggestions to generate sensory and experiential changes that modify the subjective experience of patients (Elkins, 2017), could represent significant progress in the complementary management of the physical and psychological symptoms of respiratory disease. For instance, Anlló et al. (2020) hypothesized that these perceptual modulations could be implemented to optimize breathing mechanics and reduce anxiety by suggesting a feeling of “air effortlessly entering the lungs.” There, a 15-min scripted hypnotic intervention positively impacted transient anxiety in mild and severe COPD patients (23.8% after hypnosis versus only 3% after the “relaxation and attention” control). Crucially, it also improved respiratory rate, arterial oxygen saturation, and Borg scores. We think it is plausible that this across-the-board effect may respond to the endogenously generated sensory feedback produced by the hypnotic suggestion. While promising, more evidence is needed to support this conclusion.

Wide consensus exists concerning the fundamental importance of Pulmonary Rehabilitation Programs (PRPs) for improving the clinical outcomes and behavioral patterns of COPD patients (GOLD Report, 2022). Comprehensive PRPs frequently supplement physical activity with short psychological therapy plans and self-management strategies. These have been shown to improve the psychological symptoms associated with COPD and decrease the risk of exacerbation regardless of disease severity (Coventry et al., 2013; Gordon et al., 2019). Beyond its proven clinical impact, hypnosis could greatly help with the logistic and implementational limitations that encumber PRPs (Ranjan et al., 2021). Hypnosis does not need hefty material or technological investments, its implementation is fast, and patients can obtain clinically significant relief even after short sessions (Anlló et al., 2020). Further, recent efforts assessing the feasibility of online PRPs have produced encouraging results (Beatty and Lambert, 2013; Ranjan et al., 2021). Given how hypnosis is also effective when administered through recordings and online, this renders it a suitable complement to this approach (Flynn, 2019). Additionally, most implementations of hypnosis eventually transition into self-hypnosis (Barabasz et al., 2010), which makes it a potentially useful technique for the self-management of COPD symptoms (Lenferink et al., 2017).

Given this array of advantages, we propose that an understanding of how the effects of hypnosis and self-hypnosis interact with COPD-related breathlessness, anxiety, and depression is worth considering. In particular, in patients who manifest a strong preference for drug-free approaches or have a mitigated response to pharmacological strategies. It could also be advantageous for patients who present an inability to exercise or to relax by their own means. Of course, further research in the form of new RTCs is needed before hypnosis can be endorsed conclusively for the complementary management of anxiety and depression in COPD: we still know little about hypnosis’ effectiveness across levels of disease severity, its interaction with lung function, its interaction with antidepressants, and patients’ willingness to adhere to a hypnosis-based treatment. However, given the existing evidence and current challenges in the treatment of COPD, we conclude that the effort of answering these questions is clearly justified.

## Author Contributions

HA, FL, and BH conceived the outline of the article and determined which were the important aspects to be covered in this mini-review. HA wrote the manuscript under the supervision of BH. HA and BH conducted the qualitative literature search. FL provided additional feedback and evaluated the feasibility of the review. All authors reviewed the manuscript, contributed with hands-on amendments and critical feedback, and validated the final draft.

## Funding

This study was funded by the Bligny Hospital Center (CHB) and a standard support grant from Helebor Foundation (Paris, France). HA’s contribution to this work was supported in part by the Department of Cognitive Studies at Ecole Normale Superieure de Paris, PSL University (ANR-10-LABX-0087 IEC and ANR-10-IDEX-0001-02 PSL).

## Conflict of Interest

The authors declare that the review was conducted in the absence of any commercial or financial relationships that could be construed as a potential conflict of interest.

## Publisher’s Note

All claims expressed in this article are solely those of the authors and do not necessarily represent those of their affiliated organizations, or those of the publisher, the editors and the reviewers. Any product that may be evaluated in this article, or claim that may be made by its manufacturer, is not guaranteed or endorsed by the publisher.
